# Microbiome of the Ocular Surface: Resident or Transient Ecosystem?

**DOI:** 10.7759/cureus.89487

**Published:** 2025-08-06

**Authors:** Fiorella Apuy Rodríguez, Melissa Chacón Quirós, María Laura Alvarado Fernández, María Luisa Alvarado Mora, Paula Vanegas Navarro

**Affiliations:** 1 Faculty of Medicine, Universidad de Costa Rica, San José, CRI

**Keywords:** allergic conjunctivitis, contact lens use, dry eye disease, inflammation, ocular surface microbiome

## Abstract

The ocular surface microbiome (OSM) is a low-density, low-diversity microbial ecosystem influenced by host and environmental factors, including age, hygiene, contact lens use, and systemic disease. Unlike other mucosal sites, the eye lacks a well-defined core microbiome, and its microbial composition varies significantly between individuals.

Advances in metagenomics have revealed that commensals such as *Corynebacterium* and *Staphylococcus *may contribute to immune regulation and homeostasis. Dysbiosis has been linked to ocular surface disorders, such as dry eye disease (DED), allergic conjunctivitis (AC), and contact lens-related inflammation, with shifts in microbial abundance and diversity. Continued research is needed to define resident versus transient species and to develop microbiome-based diagnostics and therapies.

## Introduction and background

The human body microbiota has been extensively studied across different body sites, including the skin, airways, gastrointestinal tract, and vagina. Its importance in maintaining homeostasis and its influence over different diseases is well established. However, the existence of a resident microbiome on the ocular surface remains controversial. The ocular surface microbiome (OSM) refers to the community of microorganisms, primarily bacteria, that inhabit the conjunctiva and cornea, forming a low-density, low-diversity ecosystem [1]. Research on the eye microbiome has been limited, and many of its functions remain unknown [1-3].

Historically, investigations of the ocular microbiome were based on traditional culture-based techniques [4]. New advances in molecular biology have contributed to our understanding of the microbial composition of the eye [2-3]. Metagenomics has provided broad insights into microbial communities; however, it also presents challenges and limitations [3]. Among these methods, 16S rRNA sequencing has become a crucial tool for a more detailed and comprehensive characterization of the OSM in both healthy and diseased states [2-4].

Unlike the gut or oral microbiome, the ocular surface is sparsely colonized by bacteria due to innate antimicrobial and mechanical barriers, including the tear film, blinking, and antimicrobial molecules [1,3,5]. Nevertheless, it remains unclear whether the microbes found represent a resident ocular microbiome or transient environmental colonizers [6].

Ocular homeostasis is crucial for maintaining eye health, and its disruption can influence various ophthalmic conditions, including dry eye disease (DED), allergic conjunctivitis (AC), and alterations related to contact lens use [2]. The OSM plays an important role in regulating ocular immunity and maintaining homeostasis; however, its exact role in disease pathogenesis remains to be fully elucidated [6]. Recent efforts, such as the Ocular Microbiome Project (OMP), are revealing crucial data on the OSM’s diversity and composition [7]. To date, a consistent and well-defined core microbiome of the ocular surface has not been established [2,8].

## Review

Methods

To prepare this narrative review, we conducted a literature search using reputable scientific sources, including ScienceDirect, PubMed Central, MDPI, BMC Ophthalmology, Scientific Reports, and the Journal of Medical Microbiology, as well as trusted online resources such as the American Academy of Ophthalmology (AAO) and the Ocular Microbiome Consortium. The search focused on studies published between 2014 and 2025, covering topics related to the OSM, its composition, influencing factors, and associations with ocular pathologies such as DED, AC, and contact lens-related dysbiosis.

We prioritized original research articles, systematic reviews, and high-impact publications to ensure the inclusion of scientifically valid and up-to-date information. Although this article does not follow a systematic review format, we aimed to synthesize the most relevant and recent findings to support a comprehensive and clinically oriented discussion.

Microbiome research techniques

Culture-based methods have traditionally been used to characterize microbiomes, but their significant limitations, especially in cultivating fastidious pathogens that require specific growth conditions, have led to the development of newer techniques. Over time, PCR has provided limited data. The emergence of metagenomics has greatly revolutionized the study of the OSM [9].

Modern methods, such as 16S rRNA sequencing and whole-metagenome shotgun sequencing, provide more accurate identification of variations in microbial abundance and species composition [9]. Studies using 16S rRNA sequencing have been more efficient than traditional culture methods, revealing greater microbial diversity. Nevertheless, this method cannot detect viruses and fungi, which are also important components of the microbiome [9]. In contrast, RNA sequencing (RNA-Seq) allows the detection of viruses and fastidious bacteria. Next-generation sequencing has further expanded research by rapidly identifying complex microbial populations, generating millions of sequences per sample [9].

Microbiome analyses commonly include assessments of alpha diversity, which reflects microbial variety within a single sample, and beta diversity, which compares microbial composition between different samples [8].

Healthy eye

The OSM refers to the population of microorganisms that reside in the human eye, specifically the conjunctiva and cornea [1,8]. The eyelids and eyelashes are considered part of the skin microbiome [5]. Although the tear film functions as a strong barrier with both antimicrobial and mechanical properties, commensal bacteria contribute to both innate and adaptive immunity [10-11].

The OSM is considered paucibacterial, with approximately 0.06 bacteria per human cell [11]. Despite this low density, metagenomic analyses using 16S rRNA sequencing have shown that while alpha diversity remains low and consistent, beta diversity is high [1,3,8]. In a longitudinal study, Zilliox et al. reported intra-individual stability over a three-month period, alongside significant variation between subjects [8].

Across studies, the dominant phyla consistently identified include Actinobacteria, Proteobacteria, and Firmicutes [2,3,8,10-11]. Zhou et al. reported that these phyla accounted for 46%, 24%, and 22%, respectively, using deep sequencing with Dacron swabs [12]. Similarly, Matysiak found that Firmicutes predominated in surface microbiota, while Proteobacteria were more abundant in deeper tissue swabs, suggesting a potential anatomical organization of microbial communities across the ocular surface [3].

Most authors agree that the genera most consistently identified include *Staphylococcus, Corynebacterium, Acinetobacter, Pseudomonas, Propionibacterium,* and *Streptococcus* [1-3,12]. Specifically, in the conjunctiva, swabs regularly reveal skin-derived bacteria, primarily coagulase-negative *Staphylococcus, Propionibacterium, Corynebacterium, *and *Streptococcus* [4,10]. Gram-negative bacteria, such as *Haemophilus, Neisseria, *and* Pseudomonas* species, have also been cultured [8].

Greater diversity has been revealed through high-resolution sequencing and culture-based techniques, identifying genera such as *Bradyrhizobium, Brevundimonas, Aquabacterium, Sphingomonas, Streptophyta, Methylobacterium, Enhydrobacter, Bacillus, *and* Ralstonia *spp. [3-4]. These findings suggest a more complex microbial population that varies depending on sampling depth and methods.

Among these, *Corynebacterium* is the most consistently prevalent and has demonstrated a protective role in neutrophil recruitment and defense against pathogens [6,10]. Notably, Ozkan proposed that *Corynebacterium* may be native to the conjunctiva rather than derived from the skin or external environment, suggesting it serves as a key component in maintaining ocular health [10].

Ocular epithelial cells appear to tolerate commensal organisms such as *Staphylococcus epidermidis *and *Propionibacterium* without initiating an inflammatory response. In contrast, exposure to pathogenic species like *Pseudomonas aeruginosa* activates an inflammatory cascade, illustrating the eye’s ability to distinguish between non-threatening and harmful bacteria [11].

**Figure 1:**
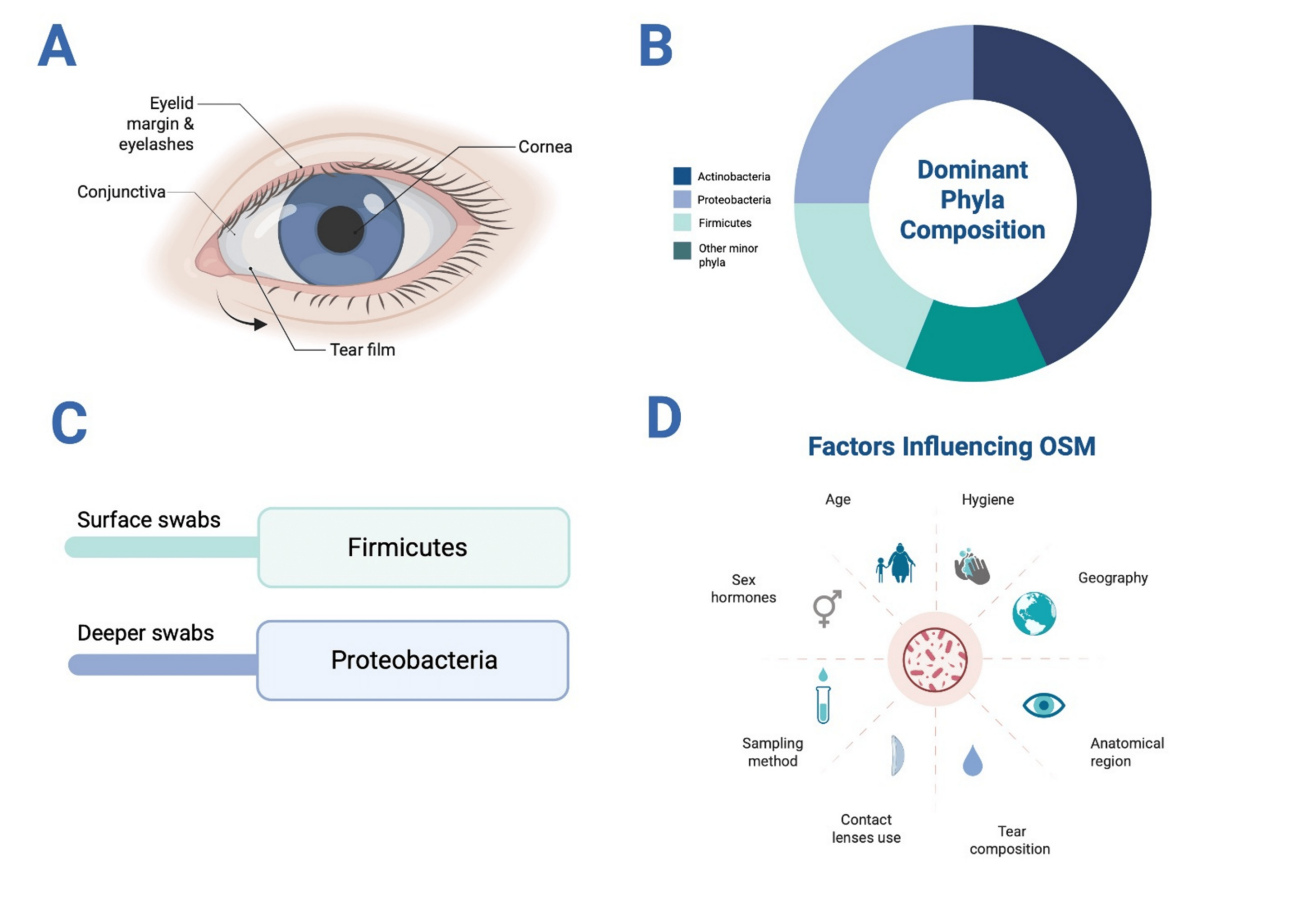
Ocular surface microbiome (OSM): anatomy, composition, diversity, and influencing factors A. Ocular microbiome zones and tear flow. The eyelids and eyelashes are part of the skin microbiome. B. Dominant phyla composition of the ocular surface. C. Spatial variation by swab depth: Firmicutes predominate in surface swabs; Proteobacteria in deeper swabs. D. Key factors influencing the ocular surface microbiome. This image was created by Dr. Fiorella Apuy Rodríguez using BioRender.

Various factors can alter the composition of the OSM, including age, geographic location, sampling method, anatomical region, contact lens use, and gender [1,8]. Tear composition and its alterations also impact the OSM. Proper hygiene practices, such as thorough hand washing before inserting contact lenses and avoiding harsh chemicals near the eyes, help maintain microbial balance. Adequate sleep has also been linked to promoting a healthy ocular surface microbiome. While treatments targeting OSM dysbiosis are still under investigation, these behavioral and environmental factors remain important in maintaining ocular surface health [13]. Figure 1 summarizes the anatomy, composition, diversity, and influencing factors of the OSM.

Dry eye disease

DED is a multifactorial disorder of the ocular surface characterized by discomfort, burning, itching, or chronic eye pain. It may also affect vision, impairing daily activities. Ocular immune tolerance prevents overactivation of the immune response to protect the tissue and preserve vision [14]. Several authors suggest that the OSM may contribute to inflammatory processes by disrupting this immune tolerance [1,3,10,15]. The OSM has been shown to interact with tear film components, promoting inflammation related to DED [14].

There are discrepant findings regarding microbial diversity in DED. Some studies report reduced diversity, while others suggest an increase [1,2]. Rizzuto et al. and Gomes et al. identified elevated levels of *Staphylococcus aureus*, coagulase-negative *Staphylococcus*, and *Corynebacterium*, whereas Kamdougha et al. reported a lower total bacterial load in DED eyes compared to controls. These inconsistencies may reflect differences in study populations, sampling techniques, or sequencing methods [2].

Schlegel et al. used whole-metagenome shotgun sequencing to discover a greater abundance of *Corynebacterium* *tuberculostearicum* in conjunctival samples and *Propionibacteriaceae* in lid samples compared to controls. In total, 229 taxa were identified from lid and conjunctival swabs, with *Actinobacteria* and *Proteobacteria *being the most abundant phyla, and Propionibacterium acnes as the dominant species [14].

DED related to meibomian gland dysfunction (MGD) does not show significant differences in overall microbial diversity when compared to healthy eyes, though specific changes in bacterial abundance are observed [1]. Borroni et al. reported that *Staphylococcus* and *Sphingomonas* are key genera in MGD. *Corynebacterium* is more prevalent in mild MGD and may contribute to inflammation as part of a protective immune response. *Campylobacter coli, Campylobacter jejuni,* and *Enterococcus* *faecium* were found in the meibum of MGD patients but were absent in healthy controls [15].

In autoimmune conditions, increased levels of *Corynebacterium*, *Staphylococcus*, and *Prevotella* were reported by Zilliox et al. [8]. In diabetes mellitus, reduced tear secretion is associated with greater microbial diversity and abundance [15]. Hormonal changes, particularly in menopause, have shown alterations in the OSM and increased DED susceptibility [11]. Environmental factors, such as prolonged screen use, reduce blinking, disrupting both the tear film and microbial balance, while eyeglasses may alter the OSM by shielding the ocular surface from irritants [1].

Allergic conjunctivitis

AC is a type I hypersensitivity response that can be potentially debilitating to the ocular surface. When a previously detected allergen is recognized, immunoglobulin E (IgE) activates mast cells, resulting in the release of preformed mediators that promote vasodilation, vascular permeability, smooth muscle contraction, and inflammatory cell recruitment. Patients typically present with itching, redness, and conjunctival swelling [16].

It is hypothesized that dysbiosis during immune system maturation, such as early-life antibiotic use, may influence the development of allergic diseases [16,17]. For example, atopic dermatitis has been associated with decreased microbial diversity and colonization by coagulase-negative *Staphylococcus* compared to controls. Dysbiosis of the conjunctival microbiome might also be associated with ocular allergies. However, few studies have directly linked the OSM with ocular allergies [17].

Studies have shown lower alpha diversity in AC, mainly associated with disease severity [1,11,18]. Beta diversity is also changed compared to healthy individuals, though no consistent pattern has been established in relation to severity [18]. The similarity between nasal and conjunctival microbiota in allergic patients has become an area of growing interest [1,11].

Song et al. used high-throughput 16S rDNA sequencing and metagenomic analysis to show that microbiota samples from AC patients were more centralized and resembled each other in composition. This suggests that the AC group may share characteristic microbiota profiles. *Corynebacterium*, *Staphylococcus*, *Streptococcus*, and *Propionibacterium* were persistently identified in these studies, with variation in relative abundance [16].

The most frequently identified genera include *Pseudomonas*, *Cutibacterium*, *Corynebacterium*, *Acinetobacter*, *Staphylococcus*, and *Streptococcus*. An increase in Firmicutes and a decrease in Proteobacteria have been observed in AC patients compared to healthy controls [18]. In addition, Inada et al. found *Blautia* and *Morganella* predominantly in the severe group. *Blautia* spp., which have also been identified in other inflammatory and metabolic diseases, may serve as potential biomarkers [18]. Song et al. also reported a higher abundance of *Bacillus* in the AC group [16].

Contact lens wear

Contact lenses are a known risk factor for inflammatory eye conditions, including giant papillary conjunctivitis and keratitis [1,4]. The OSM of contact lens users differs from that of non-users, showing similarities with the skin microbiota. This suggests that contact lenses may facilitate the transfer of skin-associated bacteria to the ocular surface or modify the OSM in favor of the proliferation of these microbes over the native ocular flora [1,4,11]. Lens material, mode of use, and user age are additional factors that may influence these microbial shifts [19].

Higher beta diversity has been observed in the OSM of contact lens users compared to controls. Several studies report a predominance of Proteobacteria, with increased levels of *Pseudomonas*, *Acinetobacter*, and *Methylobacterium*. These are potential opportunistic pathogens implicated in conjunctivitis, keratitis, and endophthalmitis [1,4,11]. Shin et al. identified other abundant bacterial groups in contact lens samples, including *Streptococcus* and members of the families *Oxalobacteraceae* and *Enterobacteriaceae*. Conversely, *Staphylococcus* levels were found to be lower in users than in non-users [4].

In patients with red eye and symptomatic infiltrates, Gomes et al. reported frequent isolation of *Haemophilus influenzae* among contact lens wearers [11]. Supporting this, Trojacka observed that *H. influenzae* was more frequently isolated in eyes with symptomatic subepithelial corneal infiltrates than in those with no ocular surface changes. Other gram-negative microorganisms were observed to promote the development of contact lens-associated red eye [19].

The presence of Gram-positive microorganisms on contact lenses, such as *Corynebacterium* spp. and coagulase-negative *Staphylococci*, is associated with increased susceptibility to peripheral corneal ulcers [19]. Furthermore, contact lens use may increase vulnerability to *Demodex* infestation [1]. A reduction in *Bacillus*, *Tatumella*, and *Lactobacillus* abundance has also been reported in orthokeratology lens users compared to non-contact lens users [19].

Current recommendations and future needs

Current OSM studies vary widely in sampling techniques, sequencing platforms, and bioinformatic pipelines, making comparisons across studies difficult. Consistent protocols for sample collection, sequencing, and data analysis are essential to ensure reproducibility and comparability [9,20]. Agreement on diversity metrics and identification approaches is also necessary for reliable interpretation [9].

Next-generation sequencing has revolutionized microbiome research by enabling rapid, high-throughput sequencing of complex microbial communities [9]. This advancement, however, demands powerful computational tools to manage the growing volume of data. Among NGS methods, 16S rRNA gene sequencing is particularly useful for detecting difficult-to-culture bacteria [18].

The therapeutic potential of probiotics, prebiotics, or targeted antimicrobials is an emerging area of interest, especially for conditions like DED and allergic conjunctivitis. Well-designed clinical trials are required to test the safety and efficacy of such approaches [6,3]. Oral probiotics have shown improvements in tear film parameters and symptoms in DED, while topical probiotics have reduced inflammation in animal models [21]. Understanding the mechanisms behind microbiome-host interactions will help develop more effective treatments. Future research should prioritize trials to validate microbiome-based therapies in ophthalmology [21].

A universally accepted definition of the core OSM is still lacking. Large-scale, longitudinal studies across diverse populations are needed to identify stable resident microorganisms and their functional roles in health and disease [1,15]. Additional investigations across different geographic populations and with larger sample sizes are necessary to confirm the generalizability of findings [16].

To support this effort, the Ocular Microbiome Project (OMP) was launched by the National Eye Institute in July 2023. This initiative supports five research centers across multiple universities to systematically collect and analyze OSM data. However, this field is still in its early stages, and there is much to uncover.

## Conclusions

The OSM is a small ecosystem that affects ocular health and disease. It is influenced by different factors, including age, gender, contact lens use, inflammatory diseases, and topical treatments. *Staphylococcus*, *Streptococcus*, *Propionibacterium*, and *Corynebacterium* have been proposed as key components, but it still remains unclear whether these microorganisms are permanent residents or transient colonizers. Alterations in the OSM have been linked to conditions such as DED, AC, and contact lens use, which cause changes in microbial diversity and abundance. Advances in molecular and metagenomic techniques have expanded the understanding beyond traditional culture methods, revealing greater microbial diversity and complex interactions. However, challenges remain in defining a core ocular microbiome, standardizing study methodologies, and clarifying the microbiome’s precise role.

Future research should focus on large-scale, longitudinal, and multi-center studies to identify stable microbial communities and their functions, as well as explore environmental and lifestyle factors influencing the OSM. Emerging therapeutic strategies targeting microbiome modulation, including probiotics and prebiotics, show promise but require rigorous clinical validation. Initiatives like the OMP provide critical information to accelerate progress in this field. A deeper understanding will enable the development of more precise diagnostic tools and innovative treatments to improve ocular surface health.
